# Cases Illustrative of the Treatment, Principally with Quinine, of Inflammatory and Complicated Febrile Affections; Being a Supplement to an Article in the July No., for 1844, of the American Journal of Medical Sciences; “on the Treatment of Inflammatory Affections of Malarious Districts”

**Published:** 1844-12

**Authors:** Wm. M. Boling

**Affiliations:** Montgomery, Alabama


### WESTERN JOURNAL

OF

### MEDICINE AND SURGERY.

DECEMBER, 1844.

ART. I.—*Cases Illustrative of the Treatment, principally with Quinine, of Inflammatory and complicated Febrile Affections,*—being a Supplement to an article, in the July No. for 1844, of the American Journal of Medical Sciences,—"On the Treatment of the Inflammatory Affections of Malarious Districts." By WM. M. BOLING, M.D., of Montgomery, Alabama.

CASE I.—*Pleuro-pneumonia of the right side,—commencing convalescence—relapse—Pneumonia of left lung.* R. Hines, farmer, about 23 years old, of rather a large frame, but pale, exsanguine, and cachectic appearance, had a 'bad cold,' and slight pain in right side for several days previous to morning of February 1st, 1844, at which time he had some chilly sensations, followed by high fever, violent cough, and acute pain under left clavicle, and over the lower part of right lung. On morning of 3d, before day, he had a decided chill, followed by an exacerbation of fever, and an increase in the pain and cough. Now, (4 o'clock P.M., 3d), pulse 124, moderately full; respiration 40; pain and cough as before stated; expectoration viscid, tenacious, yellowish but transparent, and streaked with blood; tongue moist and of a dirty yellowish color; bowels costive; great thirst; complains of a "sickening flattening feeling" just above epigastric region; considerable enlargement of spleen, of long standing. Over lower part of right lung, there is dullness on percussion, and bronchial respiration, together with a coarse crepitus along the upper margin of dull portion. Here also at one spot is heard a single loud sonorous râle. When lying on left side, at or near the middle of dull part, over a small space, is heard a slight friction murmur; respiration feeble in left, and upper part of right lung. He has just taken a dose of castor oil. ℞ Hydrarg. subm. grs. x., pulv. opii gr. i., pulv. ipecac. ½gr. Mix—to be taken at night after the operation of the castor oil.

4th. Pulse the same; respirtion 44; skin slightly moist and less hot; dilatation of pupils at each inspiratory effort; tongue same; pain in side more severe; dullness has advanced upwards a little; expectoration considerable, and so intimately mixed with blood as to be of a uniformly red color; no crepitating râle; has had three evacuations from bowels. ℞. Quin. sulph. grs.xxx., antim. tart. gr.i, ext. taraxaci, grs.viii., M. fiant. pil. viii. To take one of them every second hour, but two at first dose; epispastic to right side.

*Evening.*—Pulse and respiration the same; has had several thin evacutions, and has vomited, principally bile, three times; has taken four pills only. Omit them, and take two of the following immediately, and one every second hour. ℞. Quin. sulph. grs. xxiv., extract taraxaci grs. vi. M. fiant pil. vi.

5th. Pulse 70; respiration 24; coughs a good deal but expectorates no blood; several operations; tongue same as before, but red at edges. To resume the pills which were omitted yesterday—one every third hour. *Evening*, same. ℞. Quin. sulph. grs.vi., antim. tart. gr. $\frac{1}{4}$th, every fourth hour.

6th. He did not take the pills as directed last night. Pulse 92; respiration 52; pain in side much worse; tongue more coated. ℞. Quin. sulph. grs.xxiv., extract taraxaci grs.viii., antim. tart. gr.i. M. fiant pil. viii.; two of them to be taken every third hour.

*Evening.*—Has only taken, through mistake, half the number of pills directed this morning. Pulse 100; respiration 56; face flushed; has been bleeding at the nose; is again expectorating blood; complains of soreness about upper part of larynx, and pain and difficulty in deglutition. His blister prevents the application of the stethescope. Continue pills as directed this morning. Liniment ammonial to neck.

7th. About midnight I was called to see him on account strangury, and violent pain just at the orifice of the urethra; a frequent desire to urinate, and spasmodic contraction of urethra about the middle of the canal, which prevented its emission. ℞. Morph. sulph. gr.$\frac{1}{3}$d, immediately; warm bath; poultice to blister; continue pills.

This morning, pulse 90; respiration 30; strangury, &c., relieved; has vomited several times, and had two bilious evacuations; continue the pills.

*Evening.*—Pulse 88; respiration 32; expectoration still bloody; has vomited twice, and had three bilious evacuations. Continue the pills—two every fourth hour.

8th. Passed a better night; is sitting up by the fire, and insists on being allowed to continue doing so; says he is well, and objects to taking any more medicine; says he is hungry, though he has just swallowed a large bowl of soup.

*Evening.*—Has sat up nearly all the time to-day, till 4 o'clock this afternoon, when he had a rigor, followed by fever, increased cough, and severe pain in *left* side; pulse 102; respiration 40; expectorates with difficulty a considerable quantity of a yellowish semi-opaque matter, at times mixed with blood; strangury has returned; has vomited twice, and had three thin evacuations; tongue rather dry, more coated, and redder at edges. Repeat pills, two every third hour. Tr. opii gtt. xxv., immediately; apply cataplasms to blistered surface.

9th. Has had three thin dark evacuations; complains much of nausea and debility; pulse and respiration the same; dullness on percussion and bronchial respiration, with here and there a coarse loud sonorous râle from left nipple down; sounds healthy on right side, with the exception of a scattered sonorous râle, which is temporarily absent after each spell of coughing; skin hot and dry. ℞. Quin. sulph. grs.xxiv., ant. tart. gr.i., pulv. opii gr.i., massa hydrarg. grs.viii. M. fiat mass. in pil. viii. dividend; two of them to be taken every third hour.

*Evening.*—Is much the same; lost nearly all the pills, and consequently has not taken them as ordered; has had one thin dark evacuation. ℞. Quin. sulph. grs.xxxvi., antim. tart. gr.ss. pulv. opii gr.i., massa hydrarg. grs.xii. M. fiant. pil. xii.; three of them to be taken every third hour.

10th. Pulse 98; respiration 32; three evacuations from bowels; same stethescopic signs; continue pills; blisters to left side. *Evening.*—Same; two thin evacuations; continue same.

11th. Rested well all night; blister has drawn well; tongue more moist; pulse 82; respiration 28; continue same. *Evening.*—Pulse 78; respiration 26; three thin yellow evacuations, mixed with a little bloody mucus; no morbid sound in chest discoverable so far as the blister on left side will permit of an examinatton; continue same, substituting ext. taraxaci for the blue mass in the pills.

12th. Continues to improve; pulse 74; respiration 24; gums swollen; three evacuations; cough abated considerably; enlargement of spleen has almost entirely disappeared.

13th. Is still improving; has considerable appetite; no cough; no pain in side; continue pills at longer intervals. From this time he improved without interruption, his last blister alone causing some trouble, in consequence of its becoming inflamed from the application of poultices of elm bark, powdered, of a bad quality. This was speedily relieved by substituting poultices of bread and milk.

Although his fever, in all probability, if allowed to proceed unmodified by treatment, would have been of a well-marked tertian type, as indicated by the rigors on mornings of first and third; yet on the morning of the fifth, the time when the third exacerbation should have occurred, he was in altogether an improving condition. This was owing to his having, during the previous afternoon and night, been brought fully under the influence of quinine. On the night of the fifth, the pills were not taken regularly, and consequently we find an aggravation of all the symptoms on the morning of the 6th, and on evening of 6th still no improvement, and to account for it we find the same omission. Continuing now to take the pills regularly, on the morning of 7th notwithstanding the occurrence of a most distressing strangury, we find some improvement, and on the evening of the same day a very decided amendment. After the relapse, under the use of the same prescription continued regularly, with the exception of the omission caused by the loss of part of the pills, no improvement had taken place by evening of 9th. The quantity of quinine being now increased from 6 grains every third hour, to 9 grains every third hour, on morning of the 10th we find some amendment, and on morning of the 11th a very decided improvement in his condition. This is a fact which I have frequently observed in the administration of quinine for the cure of inflammatory affections. You administer it in moderately full doses, the patient improves for a day or two under the continued use of the same quantity, then comes to a stand, continues the same for several days, till some-something like a favorable crisis occurs, and he gets well; or probably after having remained at a stand for several days he begins to retrograde, and the termination may, or may not be fatal, according to the course pursued at this juncture. But at the time he ceases to improve, an augmentation of the dose hardly ever fails again to bring the patient into an improving condition. Relapses during convalescence from the inflammatory affections of the chest are not uncommon here, upon the least exposure or imprudence, and occasionally without either. Generally, however, the stomach and bowels are the organs secondarily affected, and this is more especially the case, after the free use of antimonials and mercurials combined. His cachectic and exsanguine appearance deterred me from the use of the lancet in his case.

Case II.—*Pneumonia.* March 29th, 1844. —— Edwards, a negro man, 45 years old, felt unwell, and had some pain in right side and shoulder, on the 24th and 25th, but not sufficient to prevent his continuing at his work in the field. On the night of the 25th he had a severe chill, followed by cough, high fever, and an aggravation of the pain in his side, which continued without abatement till morning of 28th, at which time he had some chilly sensations, and again an aggravation of all his previous symptoms. Now (10 o'clock A.M., March 29th,) pulse 120, moderately full but not hard; respiration 30; severe pain in right side and shoulder, much increased by a full inspiration; constant cough, attended with a scanty viscid white expectoration; a rough, harsh tubal sound is heard over a small space below right clavicle. With this exception there is no sound heard over right lung, synchronous with the respiratory movements, but a very faint, irregular sound, somewhat resembling that heard on the application of a sea-shell to the ear, is heard over nearly the whole lung; the rising and falling of the sound not at all corresponding to the motions of inspiration or expiration. The whole of the right side is resonant on percussion; puerile respiration in left lung; bowels costive; tongue rather dry. ℞. Hydr. subm. grs.x., pulv. ipecac. gr. ss., immediately, and ʒi. of the following mixture every second hour: ℞. Antim. tart. grs.iv., aquæ puræ ℥ij., M. Evening—Pulse 120; respiration 24; three evacuations; omit antimonials. To take tr. opii. gtt. xx., quin. sulph. grs.viii., immediately, and of the latter alone 8 grains every third hour.

30th. Pulse 100; respiration 32; complains of a very sharp and severe pain just below right axilla, and of feeling unpleasantly cool. Omit quinine, and resume antimonial, ℨj., every second hour. Evening.—Pulse 136, small and weak; respiration 56; has had four dark thin evacuations; complete dullness on percussion, from two and a half inches below right clavicle downwards; bronchial respiration, more distinct over upper three inches of dull part; just under clavicle, a very harsh puerile respiration, and the same in left lung; change of position produces no change in the extent or location of the dullness; tongue parched. ℞. Quin. sulph. ℈iiss. div. in parts V. equal; one to be taken every third hour. Tr. opii gtt. xxv., immediately.

31st. Pulse 110, weak; respiration 40; other symptoms slightly improved; continue the quinine. Evening.—Pulse 101; respiration 36; tongue slightly moist; one evacuation; continue the quinine, grs. viii. every third hour, in ℥j. of infusion of serpentaria. Tr. opii gtt. xxv., immediately.

April 1st. Pulse 80; respiration 22; tongue rather dry on dorsum, but moist at edges; continue same; blister to side. Evening.—Pulse 70; respiration 22; tongue a little more dry; through mistake the blister was applied by the nurse to the wrong side; continue same; blister to right side.

2d. Pulse 64, moderately full and soft; respiration 22; sounds on percussion and auscultation the same, with the exception of a crepitant râle heard along the line of junction between the dull and resonant part; continue same, every fourth hour. Evening.—Pulse 66; respiration 22; continue same.

3d. Pulse 64; respiration 20; dullness does not extend quite so high up; continue same. Evening.—Tongue quite moist; dullness reaches less high; otherwise he is the same; continue the same.

4th. Pulse 57; respiration 20; a loud coarse mucous râle in upper part of right lung, removable temporarily by expectoration; dullness less extensive; continue same.

5th. Pulse 60; respiration 18; dullness still less extensive; cough very much abated; tongue moist and clean; a tolerably craving appetite; continue the same; allow a little chicken soup.

6th. Pulse 54; respiration 20; scarcely any dullness; continue quinine grs. viii., every sixth hour.

The quinine was continued till the 10th. He seemed then entirely well, and it was omitted; but on the 11th a renewal of the febrile symptoms occurring it was resumed in doses of 5 grains three times a day, which was found sufficient to control the pulse. On the 15th he was going about.

A phenomenon occurred in this case which I do not recollect to have seen noticed by any author on diseases of the lungs, though it is not of unfrequent occurrence in this climate—I allude to the resonance of the chest, with the absence of the respiratory murmur or crepitating râle, preceding a rapid hepatization of the lung. The faint sound which replaced the respiratory murmur, created the idea of its production by an irregular, but very gentle undulatory motion of the residuary air alone; and this, together with the resonance of the chest on percussion, as being produced, in the first case in which I observed it, by a complete mechanical obstruction of the bronchus leading to the affected lung. The subsequent progress of the case, and my experience in others, created this impression.

Certainly in the above case the action of respiration was not attended by the entrance of air in sufficient quantity to the right lung, to produce the ordinary sounds of respiration, though at the same time percussion gave no evidence of an impermeable state of the organ. It is not unreasonable to suppose that the pain in the right side produced an inactive state of the muscles of respiration, but I observed no difference in the motion of the two sides. A kind of paralysis, or complete suspension of the function of the part seems to have existed without any appreciable physical cause in the organ itself. Are the air cells themselves and the smaller bronchial tubes so passive in the performance of the act of respiration as some pathologists suppose? I have thought that this was the first stage of pneumonia of Dr. Stokes, but it does not accord with his supposition, that that stage is indicated by "an intense puerility of respiration in the affected parts." Indeed I may say, that in this country, in at least one-half of the cases of pneumonia, a pure crepitating ràle is never heard by the physician, to precede the occurrence of hepatization. This may be partly attributable to the rapid progress of the disease, and the consequent shortness of the second stage (of Stokes) of which this ràle is indicative, thus allowing the physician less chance to discover it. This, however, is not the only cause, for in some cases marked by this absence of the respiratory murmur, in the whole or part of a lung, together with resonance on percussion, I have noted the gradual progress of hepatization almost hourly, without ever discovering a crepitating ràle. This is not the case, however, with the decline of the solidification, for this is generally followed, as it descends, by a coarse crepitous or fine mucous ràle. This form of pneumonia is I think less controlable with quinine, or indeed by any method of treatment than that which answers to the ordinary description of the disease. This is precisely the case when the hepatization, preceded by the absence of the respiratory murmur, or crepitating râle, commences in the upper part of a lung. Under such circumstances, though I have seen some three or four cases, and one of them at least in a robust and previously healthy young man, I have never known, so far as I can now recollect, a case of recovery.

If in this form of pneumonia, in which sudden hepatization takes place, not preceded by the usual signs, supposing the opinion of Dr. Stokes and others to be correct, that it is indicative of a broken down constitution or typhoid state of the system—quinine were found peculiarly applicable, it might favor the old (shall I say passing into the obselete?) opinion of its stimulant action. This, however, is not the case, at least in this climate; for while its curative agency is promptly manifested in those cases in which the ordinary routine of symptoms is observable, it is a much less efficient remedy in the other form. This insufficiency however it only partakes with the other most approved remedies for pneumonia; for whatever may be the case elsewhere, this rapid solidification, and especially when preceded by the absence of the respiratory murmur, with a resonant state of the chest —or of the crepitating râle—is as often indicative of a rapidly progressing, but highly inflammatory state, as of a different condition. It may be said that these latter remarks do not strengthen my views of the modus operandi of the remedy; be it so; they are nevertheless true, and do not militate against them.

Case III. March 30th, 1844. Diana, my own cook, a stout, healthy negro woman, about 25 years old, and six months pregnant, came to me this evening, complaining of a severe pain in right side, which she says she has had for about 48 hours. On examining I found her with considerable fever; pulse 120; some cough; constipation of bowels; tongue rather dry, and covered with a white fur, through which are apparent large and lividly red papillas. I sent her to bed and ordered a dose of castor oil.

31st. Pulse 110, small and corded; extremities rather cool; body hot; had some chilly sensations early last night; one evacution; cough almost incessant during the whole night; expectoration tenacious, clear and frothy, sometimes yellowish but transparent, and sometimes intimately mixed with blood; pain of a 'sharp teazing' character, and so severe that she can scarce refrain from crying aloud; lies on affected side nearly all the time, the pain being more severe when she lies in any other position; respiration 30; crepitous râle, at one spot marked by a friction sound, over lower two-thirds of right lung. ℞. Hydrarg. subm. grs. xx., pulv. opii., pulv. ipecac. aa. gr. i. M. to be taken immediately. Sinapisms to extremities. In an hour I bled her to 16℥, after which her pulse increased in frequency to 120, but without losing any of its force; blood thickly buffed. Ol. ricini ℥iss., immediately. Noon.—Has expectorated during the last two hours, more than a pint of tenacious mucus, so intimately mixed with florid blood, as to give it a uniform rich pink color; cough incessant, every other or every third effort being followed by the expectoration of a large mass of the above described mucus; seems drowsy, but can't sleep on account of the cough. ℞. Antim. tart. grs. iv., morph. sulph. gr. i., mucilag. acac. ℥viii. M. ℥i. to be taken every third hour. V.S. to 6 ounces; blood very much buffed. Evening.—One very large evecuation; cough and expectoration slightly diminished; pulse 126. 8 o'clock.—Pulse 128; respiration 32; expectoration diminished; pain very severe; one large thin yellow evacuation; no resonance. ℞. Pulv. opii grs. ii., antim. tart. gr. ss., quin. sulph. grs. xii. M. To be taken immediately; continue antimonial mixture ℥i. every 4th hour, and between each dose 8grs. of quinine.

April 1st. Pulse 108, fuller and softer; respiration 24; can for the first time lie on left side; tongue still rather dry; vomited several times during the night; thirst considerable; pain diminished; ràle less extensive; continue same. Evening.—Pulse 114; respiration 28; says she is much better, and has just drank, with relish, a cup of tea; complains of roaring in the ears and deafness; tongue less dry; skin nearly natural; omit quinine; continue antimonial mixture, ℥i. every 4th hour.

2d. Pulse 105; respiration 24; tongue same; continue mixture. Evening.—Pulse same; respiration 26; no resonance or vomiting; no appetite; thinks she feels worse; continue the mixture.

3d. Pulse 120; respiration 24; cough and pain in side much increased; expectoration viscid, but bloody; tongue moist, brown on dorsum and red at edges, continue mixture. Evening.—Pulse and respiration the same, pain in side still worse. ℞. mass. hydrarg. grs. xxiv., antim. tart. grs. ii., digitalis grs. iii. M.; fiant. pil. vi., one of them to be taken every third hour.

4th. Pulse, respiration, &c., the same. ℞. Mass. hyd. grs. xxxii., digitalis grs. viii., antim. tart. grs. ii. M.; fiant pil. viii., one to be taken every third hour. Noon.—Pulse and respiration the same; cough and pain much worse; v. s. to ℥xii.; blood very much buffed; continue pills. Evening. Pulse 122; respiration 30; cough and pain still increase; crepitous râle over a considerable portion of the right lung; continue the pills, one every 6th hour, and with each grs. viii. of quinine.

5th. Has taken two portions of quinine; cough and pain in side the same; four consistent evacuations; pulse 120. ℞ Quin. sulph. grs. xxxii., mass. hydrarg. grs. viii., antim. tart. gr. i. M.; fiant. pil. viii., two of them to be taken every 3d hour. Tr. opii gtt. xxv., immediately. Noon.—Has vomited several times, and had two small evacuations; is quite restless; tongue becoming redder at edges, dark on dorsum, and more dry and pointed; pulse 120, and soft; heart throbs violently. To be cupped to ℥vi. from chest; mucilaginous drinks; continue pills. Evening.—Is quite deaf; two evacuations; pulse 108; respiration 24; less pain; cough diminished. ℞. Quin. sulph. grs. xxxii., antim. tart., pulv. opii. aa gr.i. mass. hydrarg. grs. xii. M., fiant. pil. viii., two of them to be taken every third hour.

6th. Vomited several times during night; cough and pain a good deal relieved; tongue same; some florid blood expectorated last night; deafness and roaring in ears; pulse 80; respiration 18. ℞. Quin. sulph. grs. xxiv., mass. hydrarg. grs. viii. M., fiant. pil. viii., two of them to be taken every third hour. Evening.—Pulse 100; respiration 20; has had four evacuations, attended with considerable griping. To have at bed-time 8 grains of quinine, and gr.⅓ of morphine; and early in the morning 12 grains of quinine.

7th. Pulse 100; one evacuation; cough and pain much relieved; feels, she says, much better; has some appetite. To have 12 grains of quinine three times a day.

8th. Pulse 80, and in all other respects she is much improved. Continue same.

On the 10th she was able to sit up, under a continuance of the same treatment.

On the 12th, in consequence of indulging in some improper article of diet, she had a slight relapse, attended with diarrhœa and griping pains in abdomen, and a slight renewal of pain in side and cough. For the diarrhœa she took some small doses of blue mass and opium. For a slight cough, which continued to the 20th, attended at times with pain in side, she used an anodyne cough mixture, and applied tartar emetic ointment freely; while her diet was more carefully attended to.

Case. IV.—*Pneumonia.* W—— M——, a little boy, four and a half years old, was attacked, on 23d of March, 1844, with scarlatina, with which he was so slightly affected, as not to be compelled to keep his bed an entire day. About the 6th of April he became anasarcous over the whole body, and on the night of the 11th was taken with high fever, pain in chest and violent cough, which continued unabated till night of 12th, at which time I saw him, and found him with a hot and dry skin; pulse 150, hard and moderately full; respiration 72; the completion of each inspiratory effort attended with an expression of pain; tongue moist and white; thirst excessive; nausea and vomiting; expectoration scant and bloody; his restlessness and the constancy of his cough prevents anything like a stethoscopic examination. His father (a physician) has just administered grs. x. of calomel, which he immediately rejected. ℞. Hydrarg. subm. grs. vi., div. in chart. iii., one every second hour, each one, if nausea continues, to be preceded by the application of a sinapism to epigastrium and lower part of chest, for a few minutes; v. s. to ℥vi.

13th. Pulse same; respiration 50; one evacuation. ℞. Quin. sulph. grs. xv., div. in chart. v., one of them to be taken every third hour. Evening.—Pulse 130; respiration 40; continue quinine every fourth hour, and ℞ magnes. sulph. ℨi, magnes. calc. ℈i. M., to be taken immediately.

14th. Pulse 110; respiration 30; seems quite cheerful, with scarcely any cough or preternatural heat of skin; omit medicines. Evening.—Several evacuations; pulse 130; respiration 40; cough and pain increased. Resume quinine grs. iii., every third hour.

16th. Pulse 120; respiration 30, regular and easy; skin moist; continue same.

16th. Pulse 100; skin cool and moist; respiration natural; tongue moist and clean; no thirst. Under a continuance of the quinine at gradually increased intervals for 24 hours longer, he continued to improve, and was discharged well in a short time.

Case V.—*Pneumonia.* March 6th, 1844, 5 o'clock, P.M., Ashley, a stout negro man, 35 years old, has been recently exposed in some inclement weather, and in his occupation as 'ginner' for his master's plantation during the fall and winter, to the inhalation of the dust from the cotton while ginning; from which latter cause he has had a dry troublesome cough for several months.

On night of the 4th, he had a rigor, and another on morning of 5th, since which he has had considerable fever, pain in head, for which his neck has been blistered, severe and deep-seated pain in right side, and an aggravation of his cough. His bowels have been rather loose for several days, and have been still further disturbed by a large dose of oil administered this morning, which has operated profusely, producing thin, grass-green evacuations, attended with griping pains, and mixed with some bloody mucus; abdomen tender; tongue broad and moist; nausea; pulse 96 and small; respiration 32; dullness on percussion in right side posteriorly from seventh rib down, and in places a scattered dry sonorous ràle on a full inspiration; expectoration very tenacious, white and frothy; spleen enlarged. ℞. Hydrarg. subm. grs. xii., pulv. opii grs. iss. M. and div. in chart. iii., one every fourth hour; sinapisms to be followed by cataplasms to side and abdomen; mucilaginous drinks.

7th. One evacution; cough and pain in side increased, an obscure crepitating ràle a short distance above seventh rib on right side; pulse and respiration the same; dilatation of alæ

nasi at each inspiration. ℞. Quin. sulph. ℈iiss. div. in part v. equal, one to be taken every fourth hour, and in each interval an eighth of a grain of tartar emetic. Evening.—Pulse 74; respiration 28; has vomited once, and had two evacutions; tongue dry, and covered with a white fur. ℞. Hydrarg. sub. grs. v., pulv. opii grs. iss. M., to be taken immediately; continue quinine grs. viii. every third hour; omit tartar emetic; continue cataplasms.

8th. Pulse 76; respirations 30; no dullness; no sonorous, but still a deep-seated and very obscure crepitating râle; pain continues, but says generally he feels much better. Continue quinine every fourth hour; blister to side.

9th. Pulse 74; respirations 30; blister has drawn well; cough diminished; no appetite; complains much of roaring in head, deafness, and an increase of the pain in head; several operations; continue quinine in but half the quantity. Tr. opii gtt. xxv. at bed-time.

10th. Pulse 80; respirations 32; pain in side increased; tongue very much furred and red at edges; teeth dry; expectoration yellowish and in large round masses; head better; resume quinine grs. viii. every fourth hour.

11th. Pulse 60; respirations 30; one evacuation; cough and pain in side diminished; expectoration free; some appetite; continue quinine.

12th. Pulse 62, and soft; respirations 28; tongue still furred and red at edges; over a space twice as large as a dollar, near inferior angle of scapula, there is a dullness on percussion, and bronchial respiration, though both *below* and above, the sounds are healthy; has had one evacuation; some appetite. ℞. Quin. sulph. ℈iiss., ext. taraxaci ʒxii. M. fiant. pil. xii., two of them to be taken every sixth hour; repeat blister to side.

13th. Pulse 54; respirations 24; less dullness; continue quinine at gradually lengthened intervals. In 24 hours all treatment was suspended with the exception of tartar emetic ointment to side. This was continued eight or ten days, as during that time he continued to have a slight cough.

In the last four cases, as in the first, it will be observed, that at some period of the disease, before the establishment of perfect convalescence, in each case, the omission, or diminution of the quantity of quinine was followed by an aggravation of the existing symptoms, and that in each in a reasonable time, the patient was again brought into an improved condidition, by its resumption, or by an increase in the quantity.

Case VI.—*Pneumonia, &c.* September 16th, 1844, ——, a bachelor, 45 years old, of rather intemperate habits, after having had a 'bad cold and feeling feverish' for several days, was taken on afternoon of 15th with a rigor, followed by high fever, cough, and difficult respiration, but with no well defined pain in chest. He thinks his fever abated a little about midnight, but rose again about 10 o'clock this morning. Now (8 o'clock, P.M., 16th,) pulse 135, soft; respirations 44; effected principally by abdominal muscles, and diaphragm; the chest, more especially on right side, remaining almost entirely motionless; slight dullness on percusson over whole of right lung, sonorous râle at distant points heard in both lungs; puerile respiration on left side; a coarse deep crepitus is heard at some points over upper portion of right lung; tongue broad, with a dry streak in the centre; nausea and vomiting; bowels costive. He is at times slightly delirious. ℞. Quin. sulph. grs. xxxii., extract taraxaci grs. viii. M. fiant. pil. viii., to be commenced with at 10 o'clock to night, and two of them to be given every fourth hour; calomel grs. xv. immediately; sinapisms to chest and epigastrium, to be followed by a poultice.

17th. Had three consistent evacuations. A meddlesome fellow interfered with the administration of the medicine, and but two doses of quinine have been taken. Pulse 100; respiration 30; dullness scarcely if at all apparent; a coarse crepitus at points over the whole of right lung, and also a very *bass* sonorous râle during expiration; skin moist; tongue less dry on dorsum; scarcely any delirium. To have grs. xii of quinine immediately, and grs. viii. in four hours, after that grs. iv. every third hour. In case the nausea and vomiting return, morph. sulph. gr⅓d.

18th. Pulse 86; respiration 24; skin cool and moist; has not taken the medicine regularly, but in all, in last 48 hours, has taken about 36 grains; respiration still puerile in left lung; crepitous râle continues in right; complains of deafness and roaring in ears; took one portion of morphine. ℞. Quinine sulph. ℈iiss., extract taraxaci grs. xii. M. fiant pil. xii., two of them to be taken every third hour till three doses are taken, after that, one every third hour.

19th. Still improving; has some appetite. His recovery was rapid. I visited him no more.

It will be observed that all the cases of pneumonia here detailed were of the right lung, inflammation occurring in only one instance in the left, and that being a secondary attack. They all occurred near the same time, and during this period among many other cases of pneumonia besides the above, and other inflammatory affections of the thoracic viscera, I met with but one or two in which the left side of the chest was the one principally affected. The course of this epidemic tendency to inflammation of the contents of the right, in preference to those of the left side thorax, I will not attempt to explain. There is certainly something curious in it, and worthy of further observation.

Case VII.—*Scarlatina Anginosa—Laryngitis.* March 26, 1844. F. Henson, a mulatto girl, 18 years old, has been fatigued a good deal of late sitting up with a sister and child, both of whom died within a short period of each other of scarlatina maligna. On night of 18th, she felt a little unwell, and had some headache, and on the 19th she had fever and sore throat, though she continued to work as usual at the wash-tub all day. This morning early, she thinks she had a chill; pulse 134; throat very sore; tonsils and palate much swollen and vividly red, the latter dry and apparently covered with an eruption; excessive soreness about larynx, and pain on least pressure of thyroid cartilage; can't speak above a whisper; cough harsh and ringing; no expectoration; no evidence of disease of the lungs or bronchia; tongue dry and clean, with very prominent and red papillæ; bowels costive. R. Hydrarg. subm. grs. xx., pulv. ipecac. grs. ii. M. and div. in chart. iv., one to be taken every second hour. Evening—Pulse 140; two evacuations from bowels; other symptoms the same. R. Hydrarg. subm. grs. v., pulv. ipecac. grs. x., Mix, to be taken immediately. Quinine grs. vi. every second hour from midnight. Tr. Iodine to be painted over larynx externally.

21st. Pulse 130; tongue of a deep purple color, and much swollen; vomited several times during night, and had three large thin evacuations; considerable nausea, and pain in abdomen. R. Quin. sulph. grs. xxxvi., extract taraxaci grs. xii. M., fiant. pil. xii., two to be taken every second hour. Sinapisms to abdomen. Morph. sulph. gr.⅓d, immediately. Evening.—Pulse 128; six small thin evacuations; a very slight eruption on breast, scarcely perceptible; other symptoms the same; continue the quinine pills, two every third hour. Tr. opii. gtt. xx. immediately, and ten more after each evacuation; mucilaginous drinks.

22d. Pulse 124; two evacuations; less cough and soreness of fauces and larynx; papillæ on tongue as large as the head of a small pin; eruption not more perfect than yesterday; can speak a little more distinctly; continue the quinine. Evening.—Pulse 120; three evacuations; slight abatement in all the symptoms; continue the quinine.

23d. Pulse 100; tongue moist; two evacuations; has some appetite; an abatement in all the symptoms except hoarsness and pain on pressing larynx. Continue quinine every sixth hour.

24th. Still improving; pulse 90; much less hoarseness; tongue still rather red; omit medicines.

26th. Is up and doing well; cuticle desquamating; the thick skin covering the soles and lateral parts of feet, and under surface of the toes, has peeled off, in one piece from each foot, leaving the feet so tender that she can scarcely stand on them.

It will be observed that there was a gradual amendment, and abatement in the frequency of the pulse from the commencement of the use of the quinine on 20th till 23d, when a very marked improvement is found to have occurred. It is merely the gradual amendment from 20th to 27th, that I claim for the quinine, and not the sudden improvement which we find on morning of 23d, as in the then prevalent epidemic scarlatina, in almost every case that recovered, a well marked, favorable crisis occurred about the 5th day. The case indeed is introduced, principally on account of the laryngeal complication.

Case VIII.—Laryngitis—Rubeola. June 19, 1844. Blackshire, a mulatto boy, 8 years old, was taken on evening of 16th, with high fever, with very difficult and sonorous respiration, and loud ringing cough. These symptoms continued; a slight abatement taking place between midnight and day, till I saw him. Now (19th, 7 o'clock, A.M.,) pulse 144, and soft; respirations 48, and so laborious that the head is thrown violently backward and forward at each effort; dilatation of alæ nasi; a harsh, grating metallic sound, loud enough to be heard at least thirty yards, apparently produced in rima glottidis, during both inspiration and expiration; cough frequent, and partakes of this same metallic, ringing character. There is upon him a vivid eruption of measles, which appeared on 17th; tongue moist but pointed, white on dorsum and red at edges; skin hot; severe pain on pressing thyroid cartilage; sleeps, or rather lays in a stupor nearly all the time. R. Antim. tart. gr. i., aqua pura ℥i. M., a teaspoonful to be taken every second hour. Evening.—Has vomited once since dark; pulse and respiration the same; continue the antimonial solution every hour. Larynx to be painted externally with tr. iodine. 10 o'clock.—Pulse 150, soft; respiration, &c., the same; has vomited once since dark. R. Quin. sulph. grs. xviii. div. in chart. vi., one to be taken every third hour; omit antimonial.

20th. Pulse 120; breaths a little better skin moist; continue same. Evening.—Has had one consistent evacuation; pulse 112; respiration 28; sat up awhile, and appeared quite cheerful. R. Quin. sulph. grs. xxiv., hydrarg. subm. grs. viii., M., and divide in chart. viii., one to be taken every fourth hour.

21st. Pulse 100; respiration much improved, but he still seems disposed to sleep a great deal; has passed one evacuation, containing two worms, in his sleep; continue same. Evening.—Pulse 100: he is sitting up, and appears quite cheerful: allow a little chicken soup.

22d. There is still something of a ringing, metallic sound in his cough: pulse 102: eruption fading: asks for something to eat: allow a little chicken soup.

23d. Is still better. In two or three days he was running about. For more than twenty-four hours I carried my instruments, and tracheatory tube in my pocket, under the impression that an operation would be necessary.

Case IX.—Bronchitis—Slight Dysentery. Semptember 3, 1844, 10 clock, A.M. S—— P——, a mulatto girl, 8 years old, had last spring a severe attack of rubeola, followed by cough, which has continued up to the commencement of the present attack. On the morning of August 31st, she had a chill, followed by high fever: severe cough: accelerated respiration, and a feeling of oppression and tightness across the chest. Her fever abated slightly on the morning of September 1st, but before day on morning of 2d, an exacerbation, not preceded by any chilly sensations, occurred. On the 2d, she took five grains of calomel. This was followed by several bloody mucous evacutions, with severe griping pains. This evening again she has taken grs. v. of calomel in combination with a few drops of laudanum. Pulse 128; respirations 28: skin hot and dry; great thirst; tongue dry, and covered with a brown coat, red at edges: great restlessness: expectorates with difficulty some very tenacious white masses of mucus: sonorous and sibilous râle heard, mingled in all parts of chest: abdomen full, and tender on pressure. R. Quin. sulph. grs.

xii., sacch. alba grs. xxiv. M: and div. in chart. vi., one to be taken every second hour.

4th. They tell me that her fever abated a little about midnight, but commenced increasing again about three o'clock this morning: rested badly: pulse 110: respirations 24: continue quinine grs. iii. every second hour. Noon.—Pulse 105; respirations 20; some moisture of skin; tongue still dry on dorsum: stethoscopic signs the same: abdomen tender. R. Quin. sulph. grs. iii., mass hydrarg. gr. i. M: every second hour. Evening.—Pulse 100; respirations 24: râles less sonorous: skin moist: continue same.

5th. Pulse 96: respirations 24: stethoscopic signs the same: was rather restless all night: abdomen full and painful: continue same: lavement of gruel. Evening.—Pulse 96: respiration 20: one dark consistent evacuation: tongue more moist and clean: râles less numerous and less sibilous: skin moist. R. Quin. sulph. grs. iv., mass hydrarg. gr. i. every fourth hour: repeat enema.

6th. Pulse, respiration, and râles, the same: three dark consistent evacuations: a small spot on inside of lip, which has something of the character of a mercurial ulcer, though her gums are not swollen, nor is there any mercurial fetor present: continue the quinine: omit the blue mass. Evening. Pulse 90: respirations 20: râles almost entirely removed: expectoration easy: one evacuation: some appetite: continue quinine.

7th. Pulse 86: respiration easy: scarcely any cough: no râle: tongue clean and moist: continue quinine grs. iv. every sixth hour.

8th. Still doing well: omit medicine. In a few days she was entirely well, and free even of the cough which had troubled her between her convalescence from the measles, and the late attacks.

Case. X.—*Dysentery.—Pleuro-Pneumonia.* May 4, 1844, afternoon: Ashley, a stout negro man, a 'field hand,' was taken on April 28th, with fever, severe pains in abdomen, and frequent bloody mucous discharges. On 29th, he had a chill, followed by increased fever, pain in right side, and cough: in addition to the previously existing symptoms. On April 1st, and 3d, exacerbations occurred without any chill or chilly sensations. He has taken calomel, laudanum and castor oil, but in what quantities I cannot learn. Skin hot and dry: pulse 100, hard and moderately full: tongue rather dry, rough and white on dorsum, and red at edges: respirations 32: cough frequent and painful: expectoration viscid, of a dirty brown color, with streaks of florid red: griping pains in abdomen and occasional bloody mucous evacuations, but not so frequent as at first: no dullness on percussion, but low down on right side posteriorly, an obscure, apparently deep-seated crepitus is heard: here also, during expiration only, a slight friction sound, partaking a little of the creaking character, is heard. R. Quin. sulph. grs. xxiv., hydrarg. subm. grs. xii., pulv. opii grs. iss. M. and div. in equal parts: one part to be taken every sixth hour; mucilaginous drinks; sinapisms to be followed by a warm cataplasm to abdomen.

5th. Pulse 80, softer; no evacuation; other symptoms continue much the same. R. Quin. sulph. grs. xxxvi., massæ hydrarg. grs. xii., pulv. opii. grs. ii. M., fiant pil. xii., two of them to be taken every third hour. Evening.—Pulse 80: expectoration increased in quantity, and of a brighter red, and very tenacious: cough and auscultatory signs the same: tongue more moist: perspiring some about head and face: one large evacuation, containing some bloody mucus; abdomen tender, but soft: continue the pills, two every sixth hour.

6th. Cough abated, and quantity of expectoration diminished: one dark and consistent evacuation: skin cool and moist: tongue moist: pulse 64: respirations 32: râle heard over a very small space: friction sound continues: seems very languid, and scarcely speaks above a whisper: has been retching a good deal: continue same: chicken soup. Evening.—Pulse 86: respirations 36: retching continues. R. quin. sulph. grs. xxxvi., mass. hydrarg. grs. xii., pulv. opii. gr. i. M. and div. in part iv., equal, one part to be taken every sixth hour: morph. sulph. grain ⅛d, immediately: reapply sinapisms.

7th. Pulse 60: respirations 28: no crepitus: no pain, and scarcely any cough: two small evacuations. R. Quin. sulph. grs. xxxvi., pulv. opii grs. iss., mucilage acac. q. s. ut fiant mass. in pil. xii., div., two of them every fourth hour. Evening.—Same: continue pills, two every sixth hour.

8th. Pulse 50: respiration 28: one dark consistent evacuation: complains now of very little pain in side: gums slightly swollen: continue same. Evening.—Same; continue the same.

9th. Considerable appetite: no pain in the side: in other respects the same; continue the quinine grs. iv. every eighth hour.

10th. Omit medicines. In this case is very strongly marked, the controling influence of the quinine over the pulse, while the condition of the respiratory organs still remained such as to keep up an accelerated respiration.

In inflammatory affections of the chest in almost every case in which the patient is brought fully under the influence of the quinine, this want of accordance between the motions of respiration and the actions of the heart, may to a greater or less extent be observed. Instead of four contractions of the heart, to every respiration, which is considered about the normal proportion, in the above case, there were, during part of the time less than two pulsations to each respiratory act, and I have occasionally seen the proportion still smaller—say one and a half to one. An increased frequency of respiration, on the administration of the remedy, however, be it observed, has no agency in producing this discrepancy, it being entirely brought about by the diminished frequency of the pulse, the respirations gradually coming down to the healthy standard, during the subsidence of inflammatory action, while the pulse, to produce this subsidence, is frequently reduced much below the healthy standard. Can anything more strong, in proof of the effect of any medicinal agent, be adduced, than this of the sedative action of quinine, that while the inflamed state of the respiratory organs is such as to keep up, and their functional embarrassment such as to require, a much accelerated action on their part, the motions of the heart may be reduced from that increased frequency, necessary to keep up the usual accordance between these two functions, to a point far even below the healthy standard! The action of the heart and arteries once brought under the influence of the remedy, and kept so for the necessary length of time, how certainly follows an abatement in the physical signs of inflammation, together with a gradual diminution in the frequency of the respirations.

The following case is one in which a fatal result followed the administration of quinine, in consequence of the remedy having been pushed too far. Let me not be understood to mean that this is the only case in which I have seen death follow its administration—on the contrary, it is like all other remedies, liable to be misapplied or to be administered in necessarily fatal cases, and like them, consequently liable to fail in producing a favorable result. The following is one of a few cases which I have seen, in which it appears to have been not the mere negative agent, failing to cure, but the active agent, in the production of a fatal termination.

Case XI.—*Bronchitis—sequel to Scarlatina.* April 9, 1843. ———, a little boy, three years old, had an attack of scarlatina anginosa, sufficiently severe and protracted to require close attention from 20th to 29th of March, at which time he was discharged. His convalescence, however, was slow, imperfect and irregular in its progress, having occasionally slight cough, variable and capricious appetite, and a feverish state of the system sometimes at night; the cuticle desquamated slowly. Up to the time of the attack of bronchitis he had not recovered his strength sufficiently to walk alone. On evening of 8th, without exposure or any assignable cause, he was taken with violent cough, soreness across the chest, and high fever. This morning, they tell me that his fever has somewhat abated. Now, pulse 124, full and soft; impulse of heart strong, and I think felt over a larger surface than natural; respirations 68; tongue clean, moist and smooth, but not red: bowels regular; face bloated; no dullness on percussion; sonorous ràle in every part of the chest, and low down posteriorly, in addition to a fine sibilous ràle; cough, a single effort about every third or fourth act of respiration, and apparently suppressed in consequence of pain; urine *not* albuminous. R. Quin. sulph. grs.ii., pulv. ipecac. gr.ss., every third hour. Evening.—Has retained but one of the powders; he is about the same; continue the quinine, and should his fever increase, the ipecac. also.

10th. Has retained two powders, and perspired a little about the head; pulse 128; respirations 56; ràles the same; continue quinine grs.ii., and ipecac. gr.¼th, every third hour. Evening.—Medicine has not been well retained; continue same, and give immediately grs.v. of hydrarg. cum creta.

11th. A greater part of medicine has been rejected; pulse 130; respirations 64; tongue moist; ràles increased. R. Quin. sulph. grs.xvi., antim. tart. gr.ss., sacch. alba grs.xxiv. M. and div. in chart. viii., one every second hour, preceded each time by the application of a sinapism to epigastrium for a few minutes. Evening.—Pulse and respiration a little less frequent; ràles less numerous; continue the same.

12th. Pulse 116, small and weak: respirations 50: no ràle: cough very much abated: skin moderately cool: two small bilious evacuations, containing some white curdy looking masses, in consequence probably of the imperfect digestion of a small quantity of milk that he drank during the night. My little patient was a very interesting child, and I had felt an unusual degree of anxiety about his case—now, however, I loooked upon him as in a fair way of recovery. I was disappointed. Continue the powders, one every third hour: allow a little chicken water.

At my morning visit, I intended calling on him again at noon, but circumstances prevented my doing so; when I saw him again at 6 o'clock in the evening, he was dying. The whole surface of his body was cold and moist: he was entirely pulseless, but perfectly rational, speaking to the last sensibly and distinctly: his features were but very slightly sunken, and gave indications not at all commensurate with the gravity of his situation, and of no physical suffering or pain. Indeed his parents, the most watchfnl and affectionate, did not perceive any alteration in his situation, and were shocked and surprised beyond measure, hardly crediting my assertion when I spoke of his danger. His hearing was not affected. I gave him a little paregoric and aromatic spirits of ammonia, which I happened to have in my pocket, and had his extremities enveloped in mustard plasters. The plasters were on but a few minutes before he complained of their burning him. I turned from the bed to get the glass containing the stimulant, not having been absent more than three or four seconds—when I returned he was dead: certainly not more than five seconds before death he spoke distinctly and rationally.

Undoubtedly his death—can there be a question of it—was produced by the depressing or sedative action of the quinine. The quantity prescribed in the morning, was not greater than I have frequently administered to younger children. But he had been debilitated by a violent and tedious attack of scarlatina and consequently was less able to bear up against the effects of any depressing agent. The tartar emetic no doubt assisted the depressing action of the quinine. In this case the patient's disease seems to have been brought under control, but the remedy being pushed beyond the proper point, an exaggeration of that peculiar influence by which it proves curative, caused the death of the patient. It is not often that the force and fullness of the pulse is gradually reduced by quinine, as it was in this case, without a much greater proportionate diminution in its frequency.

On enquiry, I found that my morning directions had been misunderstood, and that instead of every third, the powders had been given every second hour. In addition to this, in two instances during the day, the last just before my last visit, a small portion of the powders having been wasted in their administration, the loss was compensated, by giving soon after each, another full dose.

At my morning visit, there was every indication that his disease was pretty well controlled, and his system well under the influence of the remedy. To keep up this influence, a smaller quantity was required and prescribed, and but for the mistake, I have little doubt that his recovery would have been progressive from that time. Had my directions not been misunderstood, from 8 o'clock in the morning, till 6 o'clock in the afternoon, he would probably have taken in all not more than 8 grains, and this is as much as his system would have borne. As it was, however, supposing half of two of the powders to have been lost, he must have taken about 12 or 14 grains, and with it about $\frac{1}{4}$th of a grain of tartar emetic. Still I have no idea that this quantity would have been injurious in an inflammatory attack, in one of his age, previously in good health.

Case XII.—*Tertian Remittent Fevers.—Enteritis.* October 15th, 1843, 8 o'clock, P.M., E—— R——, a little girl four years old, was taken on the 7th with, from what I could gather, a tolerably severe attack of tertian remittent fever, the remission however being very imperfect. A neighboring physician saw her only in the attack, but being himself taken sick, she was treated actively with purgatives, of which it took large quantities to produce the desired effect, by her parents, who resided some ten or twelve miles from town. Her operations are described as being sometimes fæcal, occasionally bilious, but oftener of late, mostly a reddish serum. By a close investigation, I find that she had a remission both on 12th and 14th, in the afternoon. Now, pulse 136, hard and small: skin hot and dry: feet rather cool, abdomen intensely hot, tender and swollen: is much disposed to sleep, and moans and mutters, and catches with her hands outstretched at immaginary objects, while asleep: tongue a little moist, but very smooth and red: great thirst: slight nausea: no evacuation for last twenty-four hours. R. Quin. sulph. grs.xii., hydrarg. subm. grs.iii., sacch. alba grs.xxiv. M. and divide in chart. vi., one of them to be taken every second hour; mild enema: warm pediluvia: cataplasm to abdomen: mucilaginous drinks.

16th. Pulse 126, softer: skin moist: two consistent evacuations: continue quinine alone, two grains every third hour. Evening.—Pulse 116: much less tenderness of abdomen: skin quite moist: less thirst: continue same.

17th. Pulse 112, soft: face flushed: skin dry: thirst increased: restless: continue same.

18th. Pulse 96, soft: tongue much less red: abdomen soft and scarcely at all tender: some appetite, and scarcely any thirst: continue quinine grs.ii. every sixth hour. In two or three days she was well.

Here, on the 17th, the regular time for the exacerbation, we see an attempt at one, if I may so express it, or one of a modified character, manifested by the flushed face, dry skin, and thirst, though the pulse was soft and less frequent than at at any previous period. This is not an unfrequent occurrence.

Case XIII.—*Fever—Convulsions, &c.* A sister of the above patient, 16 months old, was taken on afternoon of October 9th, 1843, with fever which gradually increased till morning of 10th, when she had a severe convulsion.

On morning of 15th, I saw her and learned that no abatement of the fever, no remission, had occurred since the convulsion. She lies apparently in a constant stupor, from which she can be but imperfectly aroused, tossing her hands and rolling her head about a good deal, cheeks seemingly swollen, and glossy: skin dry and hot: head very hot: tongue white and slightly moist: pulse 170, full, but compressible: pupils slightly dilated: abdomen full: has taken some purgative medicines. R. Quin. sulph. grs.iii. stat. sumend., and gr. iss., every third hour: tepid bath: cold lotions constantly, and cold douche occasionally to head: calomel grs.iv. at midnight.

16th. Pulse 165: some moisture of skin, and less stupor: continue quinine every fourth hour: continue cetera. Evening.—Pulse 150: less heat about head: skin moist and cool: continue same: mild enema.

17th. Pulse 140: is no longer stupid, and asks for drink: one large evacuation: skin quite moist: continue same.

18th. Pulse 136: several consistent evacuations: takes the breast willingly, and is cheerful and at times disposed to play: continue quinine gr.i. every fourth hour.

I did not see her again, but was informed that her convalescence was rapid.

Case XIV.—*Tertian Remittent Fever.—Convulsions.*—August 19th, 1844. Christine, a little girl, two and a half years old, on morning of 17th, had a chill followed by high fever, which abated a little last night. Yesterday at dinner, she was allowed, or rather forced to eat heartily of an indigestible mass of something called a pudding, though her fever was high at the time. This morning early, her father gave her 10 grains of calomel. About 10 o'clock, she had a violent convulsion, and during the time two large evacuations, one of them containing a lumbricus. At noon I saw her, she lay in a complete stupor, apparently incapable of swallowing, and noticing nothing: pupils contracted: both eyes turned to the right. Every half hour, or thereabout, since the first fit, she has had slight convulsive paroxysms, in which the eyes are opened and shut with great rapidity, the right corner of the mouth drawn towards the ear, and the right arm suddenly contracted and relaxed, and pupils *very* much dilated. These paroxysms last each about 10 minutes, and seem to come on simultaneously with a rumbling noise in the intestinal canal. Pulse 160, moderately full, but rather soft: head very hot.

I introduced 6 drops of laudanum into her mouth with a little water, which I believe she swallowed, applied a sinapism, afterwards a cataplasm, to her abdomen, another sinapism to her spine, and a blister to occiput, and had cold applications kept up constantly to her head: occasionally the cold douche. In a couple of hours, the convulsive paroxysms had considerably abated, and though still in a stupor, she swallowed readily. R. Quin. sulph. grs.xii., div. in chart. vi., one to be taken every third hour.

Evening.—Pulse 140: skin cooler: has had no fit for last two hours: continue same.

20th. Pulse 130: is awake and cheerful: skin moist: continue quinine every sixth hour till midnight, then repeat it every third hour: mild enema.

21st. The exacerbation *anticipated*, came on about midnight: she has had several slight convulsions and is in a deep stupor: pulse 140: head hot. R. Quin. sulph. grs.ii. every third hour: cold douche to head.

22d. Pulse 120: is playful, and asks for something to eat: has had several evacuations: continue same.

23d. No fever: continue quinine, two grains every third hour.

Case XV.—*Remittent Fever, Convulsions, &c.* September 25th, 1844, John Wiley, a lad 16 years old, having been in rather bad health a week or two previously, was taken with fever on 18th. From the time of the attack, his fever continued violent, with nightly exacerbations. He has been profusely purged with calomel and castor oil, and has suffered a great deal from pains, which he says shift about to different parts of his body, but most of the time are seated in the abdomen. Now, (9 o'clock, A.M.) pulse 128, small, corded and a little irregular: tongue rather dry, red at edges, and apparently stained on dorsum of a yellowish brown color, by a matter ejected from the stomach. He vomits incessantly almost, and apparently with very little effort throws up a very fetid, thick, yellowish brown, flocculent matter: thirst insatiable: spleen very much enlarged: skin, conjunctiva, and serum discharged from a blister on his abdomen, very yellow; has had for last three days almost constant hiccough: no evacuation from bowels for last 48 hours: skin alternately hot and dry, and cool and moist: the alternations irregular as to their duration, and the time of their occurrence. He suffers almost constantly with excruciating pains in abdomen, but mostly in the region of the descending colon, where there is considerable fullness. R. Morph. sulph. gr.⅓d, to be administered immediately after the first spell of vomiting, and as soon as the irritability of the stomach is allayed by the morphine, to take 8 grains of quinine. After which—R. Quin. sulph. grs.xxiv., morph. sulph. gr.i., mucilage acac. q. s., ut fiat mass. in pil. vi. dividend., one of them every second hour. Poultice to abdomen: to eat nothing.

26th. I could not visit him, but was informed that he seemed better: had an exacerbation, but less severe last night; continue the same, and give a mild enema occasionally till an evacuation is produced.

27th and 28th. I received the same information. The medicine, however, has not been given regularly, in consequence of a notion of his mother, that he will certainly die, and that it is cruel to harrass him with medicine. She is anxious, however, to give him purgative medicine, and solid food instead of chicken water, which I ordered for him.

29th. Was requested urgently to visit him to-day. Yesterday afternoon he was allowed, or rather encouraged to eat two plates of rice. His fever increased much, soon after eating it, and the pains in his abdomen became very severe, together with increased distension in region of descending colon; the vomiting, which had ceased on evening of 26th, also returned. About midnight he had two long and severe convulsions. Now, pulse small and irregular, sometimes for a little while hard, corded, and then soft: pupils dilated: stupor, and delirium when roused: tongue as before: great thirst. R. Hydrarg. subm. grs.xii., morph. sulph. gr.i. M. and div. in chart vi., one to be taken every third hour. Quin. sulph. grs.viii. immediately, and grs.iv. between each dose of the calomel and morphine: poultice to abdomen: blister to occiput.

30th. I learn that, comparatively, he had a comfortable night, still he had an exacerbation of fever. The abdominal pains are much relieved: he is more rational, and less thirsty: tongue very sore, from having bitten it during the convulsions. R. Quin. sulph. grs.xxxvi., morph. sulph. grs.ii., mucilage acaciæ q. s. ut fiat mass. in pil. xii. div. two of them to be taken every fourth hour: gum water only: mild enema, to be repeated occasionally till an evacuation is procured.

October 1st and 2d. I learn that he is improving: quite rational: continue the same: allow a little chicken water.

3d. Saw him to-day. He has improved a little in every respect: he still has once in every 24 hours a slight exacerbation in his fever, but it is becoming less usual and is deferred to a later period every day. It did not come on till after breakfast this morning, and from what I can learn is at its highest now. Has not had, notwithstanding repeated enemata, an evacuation for several days. Pulse 100: abdomen moderately full, but soft: scarcely any pain: tongue moist: skin and conjunctiva less yellow: spleen still somewhat enlarged. R Hydrarg. subm. grs.x., pulv. opii gr.ss. M., to be taken immediately. Quin. sulph. grs.xxiv., div. in parts vi., one to be taken every fourth hour: to-morrow morning a more active enema.

4th. Bowels open. Had a very slight exacerbation attended with some pain in abdomen this morning, (the pains in abdomen have invariably increased during the exacerbations of fever). No enlargement of spleen. The same prescription was continued up to the 8th, a gradual improvement taking place every day. On that day he had neither fever nor pain, and was discharged. His convalescence was slow but without relapse.

Case XVI.—*Remittent Fever.* July 13th, 1844. William Ward, a stout robust Irishman, a laborer on the rail road, had on 10th, a slight chill and fever: the fever lasting till about midnight; during the continuance of both the chill and fever, he had excessive nausea and vomiting of bile. At dark he took a dose of calomel which operated freely. On 11th, he was up but was feverish all day, and at night had a slight exacerbation. On 12th, during the early part of the day he was again better, but at night had an exacerbation in which he soon became stupid. Now, (13th, noon,) he is in a stupor, from which he can with much difficulty be roused for a few moments, to give incoherent and irrational answers; pupils natural: pulse 150, and so small as to be scarcely perceptible: surface of body generally above the healthy standard: head *very* hot: respirations 50: tongue moist and red at edges, but dry and yellow on dorsum: makes occasional efforts to vomit: abdomen full, and painful on pressure. It occurred to me that his fever might probably be of the tertian type, but as of this there could be no certainty, I determined to take advantage of the interval between noon and night, at which time, provided his fever should be of the quotidian type, the next exacerbation would occur; for in that case there would take place an aggravation of all his symptoms, and if not death, in all probability sufficient lesion of the brain or some other important organ, to lead to it. R. Quin. sulph. ℈iiss., mucilage acaciæ q. s. ut fiat mass. in pil. xii. dividend., four of them to be taken immediately, and two every fourth hour. Blisters to abdomen and neck, and sinapisms to extremities should they become cool.

14th. No amendment occurred till about midnight, when his pulse gradually became fuller and slower, and his head less hot, his stupor also wore off and he became rational. Before day he had three large dark thin evacuations: pulse 100, full and soft: respirations slow and deep: tongue same: skin cool: R. Quin. sulph. grs.viii., tr. cinchonæ ʒi. every second hour. Evening.—Pulse 112: skin hot and dry: is becoming rather stupid, very restless, lying in one position not over three or four minutes at a time: nausea and vomiting of a sour glairy fluid, sometimes mixed with bile. His fever commenced increasing soon after dinner time. R. Quin. sulph. grs.iv. every third hour.

15th. Continued in same condition till midnight, and then commenced improving. Pulse 86, soft: skin warm and moist: not at all restless: tongue same: sinapisms which were applied in consequence of his extremities becoming a little cool last night, have blistered: thirst continues: continue same.

Evening.—Got up and walked out to have an evacution, which was thin and dark: pulse 90: conjunctiva yellow: tongue same: thirst continues: continue quinine. Hydrarg. subm. grs.x. immediately.

Without any recurrence of bad symptoms he continued to improve under the use of the quinine, and one or two v. gr. doses of calomel, till 18th, when he was discharged. His tongue continued dry up to evening of 17h. When discharged, he was very much reduced, and was slow in regaining his strength.

October 16th, 1844.

